# Phylogenetic network analysis as a parsimony optimization problem

**DOI:** 10.1186/s12859-015-0675-0

**Published:** 2015-09-17

**Authors:** Ward C Wheeler

**Affiliations:** Division of Invertebrate Zoology, American Museum of Natural History, Central Park West @ 79th Street, New York, 10024-5192 NY USA

**Keywords:** Phylogenetic network, Parsimony, Optimization, Horizontal gene transfer, Softwired, Hardwired

## Abstract

**Background:**

Many problems in comparative biology are, or are thought to be, best expressed as phylogenetic “networks” as opposed to trees. In trees, vertices may have only a single parent (ancestor), while networks allow for multiple parent vertices. There are two main interpretive types of networks, “softwired” and “hardwired.” The parsimony cost of hardwired networks is based on all changes over all edges, hence must be greater than or equal to the best tree cost contained (“displayed”) by the network. This is in contrast to softwired, where each character follows the lowest parsimony cost tree displayed by the network, resulting in costs which are less than or equal to the best display tree. Neither situation is ideal since hard-wired networks are not generally biologically attractive (since individual heritable characters can have more than one parent) and softwired networks can be trivially optimized (containing the best tree for each character). Furthermore, given the alternate cost scenarios of trees and these two flavors of networks, hypothesis testing among these explanatory scenarios is impossible.

**Results:**

A network cost adjustment (penalty) is proposed to allow phylogenetic trees and soft-wired phylogenetic networks to compete equally on a parsimony optimality basis. This cost is demonstrated for several real and simulated datasets. In each case, the favored graph representation (tree or network) matched expectation or simulation scenario.

**Conclusions:**

The softwired network cost regime proposed here presents a quantitative criterion for an optimality-based search procedure where trees and networks can participate in hypothesis testing simultaneously.

## Background

Many problems in comparative biology are, or are thought to be, best expressed as phylogenetic “networks” as opposed to trees. The central idea being that trees convey only vertical information transfer between ancestor and descendant and networks can include reticulation (“network”) events representing horizontal transfer of information between lineages. This may be due to hybridization (e.g some plants and parthenogenetic lizards; [1]), exchange of particular genetic elements (e.g. bacteria; [2]), or exchange of chromosome-like segments (e.g. influenza viruses; [3]), among other causes.

Incongruence among data sets (usually molecular sequences) is the most frequently cited evidence for invoking networks and their multiple character histories. Molecular sequences with different histories (through horizontal exchange) would be expected to show incongruence in the form of different trees as “best” historical solutions. Unfortunately, other explanations are possible, most obviously simple homoplasy (non-minimal change of characters on a tree; [4]). It is rare to put it mildly, for non-trivial datas sets (those with more than a handful of characters and taxa) to be completely consistent. If this were not the case, there would be no cause for most of systematic theory or computational effort, since all problems would reduce to “perfect phylogeny” and allow exact, polynomial-time solutions [5].

A powerful example of this homoplasy/multiple history phenomenon is presented by [6]. In a case of whole *Vibrio* genomes, none of 243 individual locally collinear genomic regions, which yielded 286 unique topologies (the greater number of trees than genomic regions is due to multiple equally costly solutions for individual regions), agreed with their combination (whole-genome phylogeny). On the other hand, multiple random nucleotide samples of the same size as the individual loci, but drawn across the entire genome agreed in each case with the whole-genome phylogeny. The individual collinear regions are highly localized samples that yielded unique phylogenetic trees, while a single underlying signal was present throughout the genome. The distinction between actual multiple history and simple homoplasy is central to the analysis of networks and trees. The ability to distinguish between the two is a fundamental purpose of phylogenetic network analysis.

Whatever the motivating mechanism, there are many types of networks, or at least network diagrams in the literature (reviewed in [7, 8]). Some are not meant to represent historical scenarios, but summaries of conflicting phylogenetic information (e.g. “split” trees and networks; [9]), viral reassortment events (“reassortment networks”; [10, 11]), or multiple, differing trees (e.g. “cluster” networks; [12]).

The networks considered here are “phylogenetic” networks as described by [13] that strive to explain transformation events in terms of vertical and horizontal events on graphs that connect terminal (leaf) taxa to each other and to a single root as on a traditional phylogenetic tree, but with additional, network edges. Furthermore, this work only deals with networks as a parsimony problem, likelihood network methods have been proposed (e.g. [14, 15]) but are not further discussed here.

## Trees and networks

**Fig. 1:**
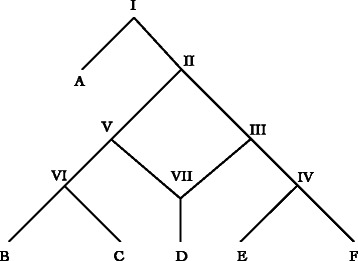
Network with leaves A–F, root node I, tree nodes II–VI, and network node VII. Edges V–VII and III-VII are network edges, other edges are tree edges

A tree is typically defined as a directed acyclic graph (DAG) with vertices (nodes) of three types: those with indegree=0 and outdegree=2 (root), indegree=1 and outdegree=0 (leaves or terminals), and indegree=1 and outdegree=2 (internal or HTU nodes) (summarized in [16]). Networks are a superset of this, allowing for reticulate (i.e. network) nodes with indegree > 1. Here the conventions and definitions of Moret et al. [13] are followed. This limits (rooted) network nodes to indegree=2 and outdegree=1, and forbids edges that directly connect network nodes. Edges that end in tree nodes are referred to as tree edges, and those that end in network nodes as network edges. Furthermore, potential network edges are constrained that they be, at least potentially, contemporaneous (no ancestor to descendent network edges) consistent with the notion of lineages exchanging information at a particular time (Fig. 1).

**Fig. 2:**
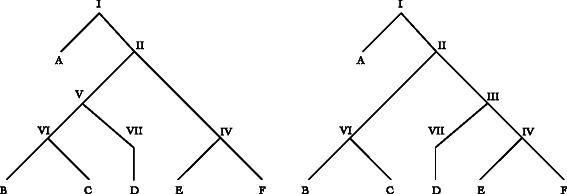
Binary “display” trees of network in Fig. 1. Node VII (now indegree=outdegree=1) can be removed by contraction

*Soft and Hard*–there are two fundamental interpretations of the meaning of phylogenetic network edges: “softwired” and “hardwired” [7]. Softwired networks and their edges represent alternate edges only one of which is found in any given “display” or resolved binary tree (Fig. 2). A softwired network with *n* network nodes will have at most 2^*n*^ binary resolutions of display trees [17]^1^.

Network edges in hardwired networks are all present and signify potential transformations between multiple ancestors and a single descendant. These alternate interpretations (soft and hard wired) lead to alternate definitions of the parsimony cost of these network types. For a network *N* with set of display trees *τ*(*N*), and a set of characters *C* to be optimized on *N*, the parsimony score of a given character *c* will be the best score found for that character on any tree *T* in *τ*(*N*). The overall softwired parsimony score, *S*(*N*,*C*) [18–20] will be :

(1) $$ S(N,C)_{score} = \Sigma_{c \in C} \text{min }_{\left(T \in \tau \left(N \right) \right) }T^{c}_{score}.   $$

One immediate problem with such cost, as pointed out by [20], is that there is a trivial minimum cost where each character is assigned its best tree. In essence, when there are many display trees in a network each character can be optimized on a tree that provides minimal cost. To overcome this, [20] recommended partitioning the character set into blocks that would be optimized on the same display tree. These blocks could be more or less subjective, based on gene sequences or other criteria.

Hardwired costs on the other hand (*H*(*N*,*C*)_*score*_) do not depend on display trees, but are the sum of the weights of all edges (*e*) in the network *N*, where the edge weights (*w*(*e*)) are the minimum number of character changes between vertex states that bound each edge [21, 22].

(2) $$ H(N, C)_{score} = \Sigma_{c \in C} \Sigma_{e \in N} w_{c} (e).  $$

The time complexity of determining the softwired parsimony score is exponential in the number of network nodes (*r*) but polynomial for non-additive/unordered [23] type characters when *r* is fixed. Determining the hardwired cost is NP-hard (but fixed-parameter tractable in the parsimony score) [24] when the number of character states exceeds 2.

Biologically, the softwired interpretation is in general more attractive in that it allows for multiple ancestor scenarios, but only a single ancestor for a given character. Scenarios of horizontal gene flow are thought to represent alternate binary tree (ancestor-descendent) scenarios, such that a given taxon might have multiple ancestors, but a given feature only one. For example, when horizontal gene transfer occurs, the ancestry of bacterial genomes can be represented by multiple independent trees, one for each set of loci that have been transferred. Even characters in hybrid origin lineages are generally thought to have a single ancestral origin, just mixed in a 1:1 ratio throughout the genome as opposed to the much smaller fraction implied by single gene horizontal transfer (this could also be said of biparental inheritance systems).

## Optimality and hypothesis testing

Given the scoring differences among softwired and hardwired networks and binary trees, it is impossible to compete them on an equal footing in a hypothesis testing framework. Softwired will always be shorter (or worst case equal to trees), and hardwired always longer (or best case equal to trees).

Due to the seemingly greater biological utility of softwired networks, the remainder of this discussion will be restricted to the issue of optimality and hypothesis testing among competing tree and softwired network (referred to simply as “network” hereafter) scenarios. Basically, some penalty, dependent on the degree of “network-ness” (defined below), must be applied, such that tree costs and network costs are comparable.

## Network edge penalty

There are several behaviors that are desirable in a network penalty. First, the penalty should be dependent on the number of extra (i.e. non-tree) edges in the network scenario, the less tree-like, the higher the cost. Second, this penalty must be applied on a character-by-character basis. Since characters can have different histories (or we wouldn’t be bothering with networks in the first place), most character state transformations may be represented by a single optimal display tree, while other character transformations may be following multiple, alternate display trees. Third, networks containing superfluous edges (those unused by any character transformations) must be assigned an infinite cost. This is to ensure that only the minimum number of edges required are identified. Otherwise, the solution to all cases would be a network that contains all possible binary trees.

The basic idea of the network penalty is to account for the “expected” change in cost as extra edges are added to a tree. The factor suggested here is that the improvement in parsimony score for a network as edges are added is $\frac {1}{2}$ of the expected cost of each edge for a tree with *n* leaves, *T*_*cost*_/(2*n*−2). The factor of $\frac {1}{2}$ is motivated from the minimum metric cost of inserting characters *de novo*, as opposed to substitution in character change on a given edge. This factor is derived from the triangle inequality setting a lower bound on the ratio of insertion-deletion events and character substitution [25]. Basically, metricity demands that that the cost of character change between states (say nucleotides adenine and cytosine) must be less that the cost of deleting one and inserting the other. If this were not the case, substitutions would never be optimal since paired insertion and deletion would always be lower cost. This requirement offers a non-arbitrary method to establish the benefit of extra (ie. network) edges. The degree to which improvements in network costs are greater than this amount determines the optimality of the network scenario.

Consider a network *N*=(*V*,*E*), as commonly defined with an edge set *E* and vertex set *V*. Furthermore, consider the set of display trees *T* derived from the resolutions of network edges in *E* with *n* leaf taxa. For a set of *k* characters *C*=(*C*_1_,…,*C*_*k*_), there is at least one most parsimonious (for all characters combined) display tree *τ* ^*min*^ at cost *c* *o* *s* *t*(*τ* ^*min*^) with edge set *E* ^*min*^ and vertex set *V* ^*min*^. Other trees in the display set *T*, *τ*∈*T* have edge sets *E* and vertex sets *V*. We further denote the display tree with minimum cost *c*_*i*_ for a given character *C*_*i*_ as *τ*_*i*_ with edge set *E* ^*i*^.

We can then define *a* (as opposed to *the*) network cost as the softwired cost (eq. 1) augmented by a penalty: *S*(*N*,*C*)_*cost*_+*P*(*N*,*C*) where

(3) $$ \begin{aligned}{} P \left(N, C \right) =\left\{ \begin{array}{ll} \frac {\Sigma_{i=1}^{k} c_{i} \times | E^{i} \setminus E^{min} |} {2 \times \left(2n -2 \right)}, & \text{if all network edges ``used''} \\ \infty & \text{otherwise.} \end{array} \right. \end{aligned}  $$

This penalty assigns a cost for each edge in the trees of minimum cost for each character (individually) not found in the overall best (for all characters) display tree with the multiplicative factor |*E* ^*i*^∖*E* ^*min*^|. Since the penalty for any tree is 0 (since there are no extra edges) and the softwired cost is equal to the tree cost, the penalty only affects the optimality of networks. *P*(*N*,*C*) is set to *∞* if any edge is “unused” in the network. Unused is here defined as an edge that is not a member of a minimal cost display tree for any character.

## Methods

### Example cases–observed and simulated

To explore the behavior of this network penalty, two biological and one linguistic data sets were employed. For the biological data, several simulated versions based on single and multiple gene history were created to further test the penalty. This demonstration is not meant to represent an exhaustive treatment of the network penalty, but an illustration of how this penalty behaves in tree-like and network-like cases.

The biological examples consist of a data set of 12 microhylid frogs and 7 loci (2 mitochondrial and 5 nuclear) drawn from [26], and an H1N1 2009 influenza data set of 9 complete genomes of 8 segments drawn from [3]. The linguistic data are the Uto-Aztecan data of 40 languages and 102 words of [27].

The two biological data sets were chosen as cases where networks were (influenza) and were not (microhylids) thought to be reasonable historical scenarios. The linguistic data set is based on words (Swadesh 100 list; [28]) thought to be less prone to borrowing (horizontal transfer), but several have been hypothesized to have undergone some exchange in subsets of Uto-Aztecan languages and exchange from non Uto-Aztecan languages that are geographically adjacent.

#### Analysis of observed sequences

For each of the three data sets, the most parsimonious (“best”) heuristic tree solution for combined and partitioned loci/segments was created using POY5 [29, 30]. The cost regime was completely homogeneous (substitutions = insertions = deletions =1) using unaligned sequences.

**Fig. 3:**
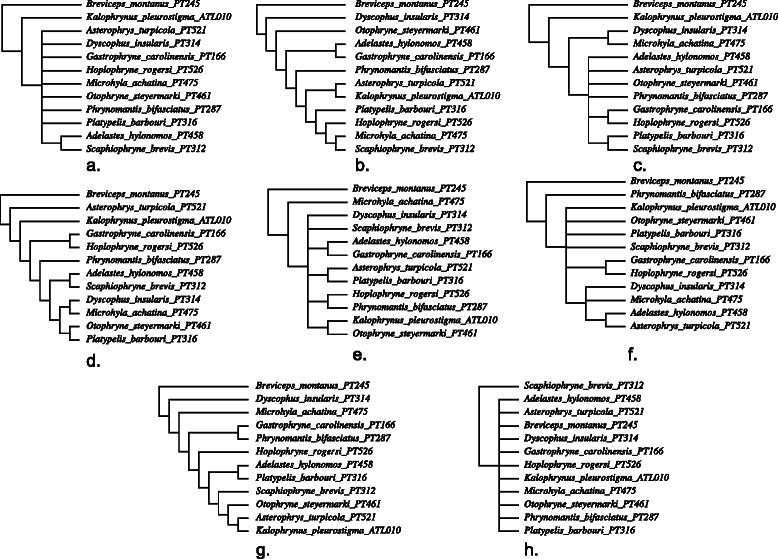
Microhylid trees for individual loci and their strict consensus (**a**–Tyrosinase, **b**–Seventh in Absentia, **c**–Histone H3, **d**–Cytochrome Oxidase 1, **e**–Cellular Myelocytomatosis Oncogene - Exon 2 (CMYC), **f**–Brain-derived Neurotrophic Factor (BDNF), **g**–16SrDNA, and **h**–strict consensus of all loci;. Data from [26]

**Fig. 4:**
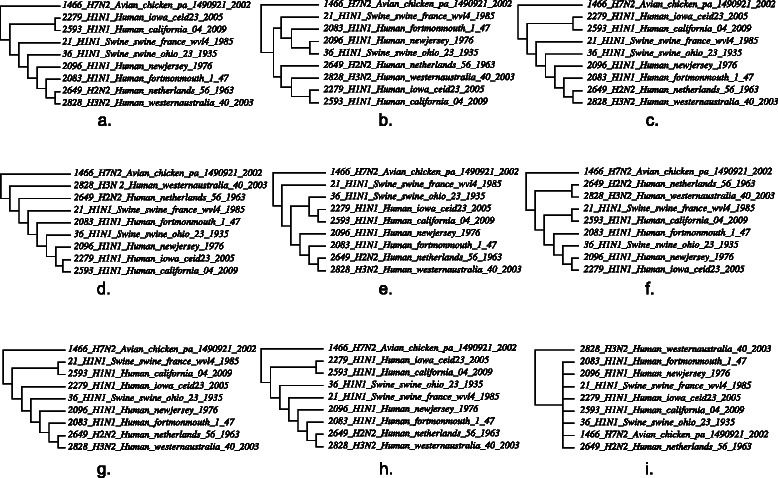
Phylogenies based on analysis of sequence data from a sample of viral isolates [3] for each segment of the H1N1 2009 influenza genome (**a**. 1 (PB2), **b**. 2 (PB1), **c**. 3 (PA), **d**. 4 (HA), **e**. 5(NP), **f**. 6 (NA), **g**. 7 (MP), **h**. 8 (NS)) and their strict consensus (**i**.)

**Fig. 5:**
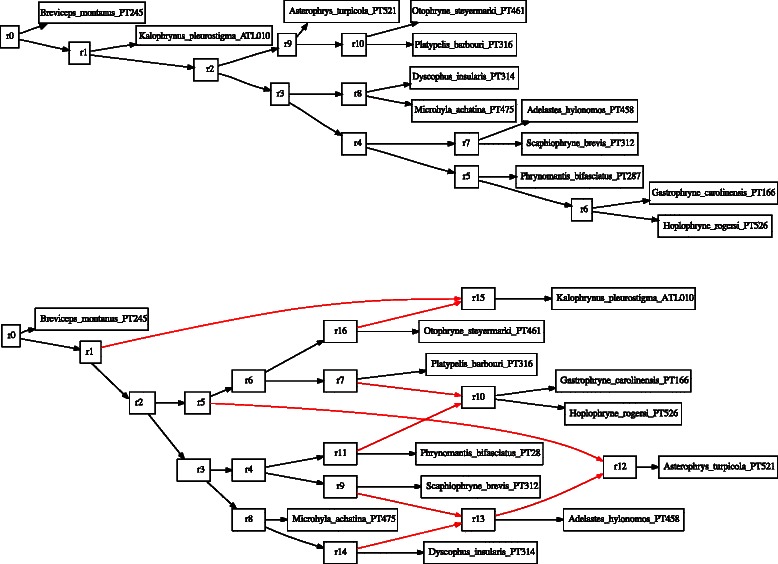
Microhylid tree (top, based on concatenated data) and network (bottom). Network edges in red. Internal vertices are labelled “rN”. Data from [26]

**Fig. 6:**
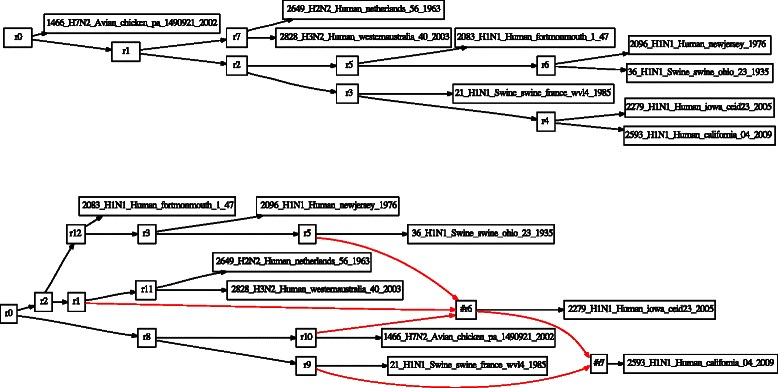
Avian influenza tree (top, based on concatenated data) and network (bottom). Network edges in red. Internal vertices are labelled “rN”. Data from [3]

**Fig. 7:**
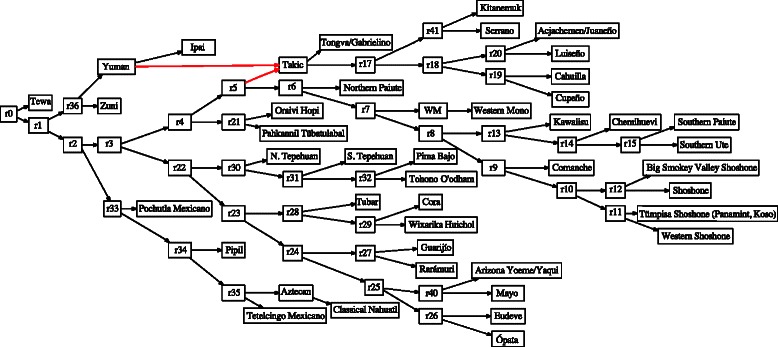
Softwired network of Uto-Aztecan languages with a network node at the base of “Takic” languages, denoting contributions from Yuman as well as Uto-Atecan parent languages (red edges). Internal nodes are labelled as “rN”. Data and base tree (without Yuman-Takic edge) from [27]

A combination of Wagner random addition sequences (100 replicates), TBR refinement, and tree recombination (fusing) [31, 32] was employed for each analysis. Partitioned analyses are shown in Figs. 3 and 4. Candidate network scenarios were created in two ways. For the microhylid data, loci were analyzed independently (Fig. 3) and edges added to the simultaneous tree solution to create the candidate network. These network edges were based on minimum hybridization networks derived using Dendrosope [33] (Fig. 5). Networks were diagnosed using a prototype network tool, PhylogeneticComponentGraph (PCG; https://github.com/wardwheeler/PhyloComGraph.git) reading fasta and extended newick [34] files using the commands read(~*.fas~) read (newick:~network.enewick~). Currently, networks can only be diagnosed from input, not searched. With the influenza data, the reassortment scenario of [3], was used for network diagnosis (Fig. 6). For the linguistic data, the base tree of [27] was used, augmented by a scenario of Yuman-Takic exchange (in loanwords suggested by Jane Hill recorded in Kenneth C. [35]) (one edge; Fig. 7). Other exchanges regarded as unlikely (e.g., Aztec–Shoshone, Western Mono–(Eudeve + Òpata)) were tested as well.

#### Analysis of simulated sequences

In order to add greater control to test cases, the two biological data sets were used as a basis for simulations using DAWG [36]. The linguistic data set was not a basis for simulation due to its large sequence alphabet. In both cases, the length and number of loci in the datasets (7 for microhylids, 8 for influenza) were simulated under three scenarios. In the first, all of the loci/segments underwent simulated evolution on the same tree with the same branch lengths as determined by the combined tree analysis in POY5 (“COM”). In the second, the same single COM tree was used but with unique branch lengths (again based on analysis in POY5) for each locus/segment (“SEP”). In the third, each locus/segment had its own tree and branch length set based on independent analysis using POY5 (“IND”). The first two cases reflect alternate scenarios of tree-like evolution, whereas the third is network-like (Table 1). For each of the 45 runs, a full GTR+G+I model ([37, 38]; rate parameters for AC, AG, AT, CG, CT, GT = {1.5,3.0,0.9,1.2,2.5,1.0}, nucleotide frequencies A, C, G, T = {0.20,0.30,0.30,0.20}, *Γ*=1, I =0.1) was used with gap model “NB” using {1,0.5} for insertions and {2,0.5} for deletions.

**Table 1:** Results of tree and network analyses of observed and simulated data for microhylid frogs and influenza virus strains. Tree cost values are the minimum of the display tree set. The simulated result procedures,“COM,” “SEP,” and “IND” are defined in the text. Values of *∞* in “Penalty” and “Network” signify that there was at least one “unused” edge in the network

Tree, network, and penalty costs					
Data set	Scenario	Observed	COM	SEP	IND
Microhylids	Tree	3962	3535	3695	4076
	Softwired	3939	3535	3695	3964
	Penalty	32.64	*∞*	*∞*	83.59
	Network	3971.64	*∞*	*∞*	4047.59
Influenza Virus	Tree	10272	8443	9169	9092
	Softwired	9935	8443	9169	8775
	Penalty	324.59	*∞*	*∞*	270.56
	Network	10259.59	*∞*	*∞*	9045.56

## Results and discussion

The results of observed and simulated analyses for the biological data sets are summarized in Table 1. Those of the linguistic analysis are contained in Table 2.

**Table 2:** Results of tree and network analysis of Uto-Aztecan linguistic data. Tree cost values are the minimum of the display tree set

Tree, network, and penalty costs				
Data set	Scenario	Yuman–Takic	Aztecan–Shoshone	WMono–Eudeve/Òpata
Uto-Aztecan	Tree	10120	10120	10120
	Softwired	10063	10118	10113
	Penalty	21.833	0.94	4.23
	Network	10084.83	10118.94	10117.23

The analyses of observed data (both biological and linguistic) show patterns that are largely as expected. The microhylid data, where horizontal exchange was not thought to occur, showed the optimal solution as a tree. The influenza data displayed the opposite behavior with (penalty adjusted), network cost superior to that of the best tree solution, indicating that allowing reassortment shows these viruses evolved not only via mutational processes (Table 1). The linguistic data showed a marked preference for the Yuman-Takic exchange scenario over both the tree alone and other exchanges not thought likely (although these showed marginal superiority-0.1 %-to the tree solution as well) (Table 2). This is particularly acute, given that, for the biological data sets, there is no non-trivial pattern of relationships shared among all loci/segments in either the microhylid or influenza data sets. Both show near complete incongruence, but show markedly different relative network optimality.

The simulated data show a series of consistent patterns. Where independent evolution among genetic elements was simulated, network solutions were favored. In the cases of single tree simulations, whether with either common or independent branch lengths, there were unused edges, hence, tree solutions were favored over networks. A point to note is the close correspondence of simulated and observed data costs (in terms of overall character change), supporting the utility of the modeled data. However, the presence of unused edges suggests that the simulations were perhaps overly “clean” in their tree-like patterns.

## Conclusions

Incongruence among sequence data (especially genetic loci) has often been seen as evidence of multiple ancestor origins of transformation. This is in opposition to narratives attached to non-sequence data (e.g.,anatomy, codon position) where disagreements among characters are ascribed to simple homoplasy (e.g., reversal, parallelism). One of the key questions to be addressed is when are such character incompatibilities indicative of multiple history as opposed to simple non-minimal change? As discussed above, incongruence among loci, even in whole-genome analysis, can be due to non-random sampling effects (contiguous sequence positions) as opposed to multiple historical signals [6].

Obviously, not all incongruence can be ascribed to multiple history, but where is the line to be drawn? That is the objective of this discussion. How can we compete network and tree solutions on an equal footing?

Given the match of expectation with observation in the biological and linguistic data, as well as the behavior of the simulated data, the softwired network cost proposed here is worth considering as such an optimality criterion. In each of the 11 cases examined, trees were favored where they were thought most reasonable and networks where they had been proposed or simulated. This success is tempered by three caveats.

First, the networks generated were not chosen based on any measure of quality. Network edges were added to (parsimony searched) trees based on hybridization networks. This is adequate to identify potential reticulation events and illustrate the behavior of the proposed network cost, but the quality of these networks (compared to others) is unknown. A more complete discussion awaits more effective network identification. Second, the test cases discussed here are limited. A broader sample of real and simulated data will be required to explore fully the behavior of any network cost. Third, although the network penalty proposed here is based on the logic of metric character transformation and softwired networks, other costs are possible. These might weight particular edge cost components differently, or have alternate expectations as to cost reductions (in comparison to trees) as networks become more complex. Furthermore, different sorts of penalties will yield different results.

Even acknowledging these concerns, the softwired network cost regime proposed here presents a quantitative criterion for an optimality-based search procedure where trees and networks can participate in hypothesis testing simultaneously. Only through such a procedure, can we address questions of the competing influence of vertical and horizontal transfer of information in evolving systems.

## Endnote

^1^ This motivates the [13] restriction that network nodes cannot have another network node as a parent. Such a situation can result if both descendants of a network node are also network nodes yielding display trees with internal vertices promoted to leaves.
